# Clinical and virological characteristics of coexistent hepatitis B surface antigen and antibody in treatment-naive children with chronic hepatitis B virus infection

**DOI:** 10.3389/fpubh.2024.1380771

**Published:** 2024-06-17

**Authors:** Yi Xu, ShuangJie Li, WenXian OuYang, Zhenzhen Yao, Xin Lai, Yingping Gu, Meng Yang, Ling Ye, Sisi Li, Songxu Peng

**Affiliations:** ^1^Department of Maternal and Child Health, Xiangya School of Public Health, Central South University, Changsha, China; ^2^Department of Hepatopathy Center, Hunan Children's Hospital, Changsha, China

**Keywords:** coexistence, biochemical indices, virological indices, anti-HBs, children

## Abstract

Serological pattern of simultaneous positivity for hepatitis B surface antigen (HBsAg) and antibody against HBsAg (anti-HBs) is considered a specific and atypical phenomenon among patients with chronic hepatitis B virus (HBV) infection, especially in pediatric patients. Unfortunately, there is limited understanding of the clinical and virological characteristics among children having chronic HBV infection and the coexistence of HBsAg and anti-HBs. Hence, our objective was to determine the prevalence of coexistent HBsAg and anti-HBs and to explore the associated clinical and virological features in this patient population. The researchers conducted a retrospective cohort study on the 413 pediatric patients with chronic HBV infection from December 2011 to June 2022. The patients were stratified into two groups based on their anti-HBs status. Demographic, serum biochemical and virological parameters of two group were compared. Of the total 413 enrolled subjects, 94 (22.8%) were tested positive for both HBsAg and anti-HBs. Patients with anti-HBs were younger and demonstrated significantly higher ratio of albumin to globulin (A/G), elevated serum levels of alanine transaminase (ALT), lower ratio of aspartate transaminase (AST)/ALT (AST/ALT) and reduced serum levels of globulin, HBsAg and HBV DNA, Additionally, these patients were more likely to show coexistent HBeAg and anti-HBe when compared to patients without anti-HBs. The results of multivariate logistical analysis revealed that AST/ALT, serum levels of globulin and HBsAg were negatively associated with coexistence of HBsAg and anti-HBs. Our data demonstrated a considerable prevalence of coexisting HBsAg and anti-HBs in pediatric patients. Children with this specific serological pattern were commonly of a younger age, seemly predisposing them to early liver impairment and lower HBV replication activity.

## Introduction

Chronic hepatitis B virus (HBV) infection poses a massive burden to global health (1). Based on a global, regional, and national study published in 2022 (1), the global prevalence of chronic HBV infection approximated an intermediate level of 4.1%, corresponding to 316 million patients. Despite the implementation of effective prevention strategies involving vaccines and therapeutic interventions, nearly 2 million children under the age of 5 continue to acquire new HBV infections annually (2, 3). Remarkably, China remains at the top of the list for HBsAg-positive infections and ranks tenth in terms of number of infected persons in children under the age of 5 (3). Infants and children exposed to HBV have a greater susceptibility to develop chronic hepatitis B (CHB) (1, 2, 4), which substantially elevated the lifelong risks of adverse outcomes linked to HBV, including liver cirrhosis and hepatocellular carcinoma. These highlight the need for more attention for pediatric patients with chronic HBV infection.

Since the discovery of an uncommon serological pattern in patients with CHB characterized by the coexistence of hepatitis B surface antigen (HBsAg) and antibody against hepatitis B surface (anti-HBs), there has been a growing interest in this phenomenon. Several studies reported that this coexistent phenomenon was closely associated with adverse outcomes including HBV reactivation (5, 6), hepatic fibrosis or cirrhosis (5, 7), and hepatocellular cancer (5, 8, 9). Therefore, it is necessary to explore clinical features related to virology of CHB children with dual positivity for HBsAg and anti-HBs to favor to identify patient population with potential risk of adverse outcomes, which allows for better maintenance and management of the health of children with CHB.

Previous studies have extensively examined the clinical characteristics and the virological features in CHB patients tested concurrently positive for HBsAg and anti-HBs (7–12). By comparing the differences between patients with and without anti-HBs in indicators including age, gender, and serum alanine transaminase (ALT), aspartate transaminase (AST), HBsAg and HBV DNA, etc., these research revealed that the patients with anti-HBs were generally older and had lower HBsAg. However, the results of HBV DNA, ALT and AST levels varied. These studies have mainly focused on adult patients with chronic HBV infection, while available data on children population is limited. Compared with adult patients, children with chronic HBV infection exhibit significant differences in terms of the natural history, immunity to HBV and serological features, as well as the risk of poor hepatic outcomes (2). Therefore, the main purposes of this study are to assess the prevalence of coexistence of HBsAg and anti-HBs in chronically HBV-infected children and depict corresponding clinical and virological features.

## Materials and methods

### Study design and patients

A retrospective cohort study was conducted in the hepatology center of Hunan Children’s Hospital from December 2011 to June 2022. Patients who conformed to the following criteria were included: (1) less than 18 years old; (2) tested positive for HBsAg continuously more than 6 months; and (3) received no antiviral treatment for chronic HBV infection before. Patients who had been diagnosed with other viral infection (including EB virus infection, cytomegalovirus infection, etc); other viral hepatitis infection (HAV, HCV, HDV and HEV, etc); non-viral hepatitis (including acute icteric hepatitis, drug-induced hepatitis, and autoimmune hepatitis, etc); other related-liver diseases (cholestasis, non-alcoholic fatty liver disease, acute liver failure, hepatic hemangioma, hepatolenticular degeneration, etc); other diseases with abnormal immune system (allergic disease, etc); thrombocytopenic diseases (hemophagocytic syndrome, leukemia, immune thrombocytopenic purpura, pancytopenia, etc) or had received any liver protection drug therapy were excluded. In addition, due to the retrospective data collecting, patients with incomplete data on key variables were excluded. This study received approval from the ethics committees of Xiangya School of Public Health Central South University (XYGW-2023-123), with a waiver of informed consent due to its retrospective nature.

### Variables acquisition and evaluation

Demographic data including age, gender, maternal HBV infection status, vaccination status were collected during hospital visits of children. Serum biochemical indices were measured using Bayer 2,400 automatic biochemical instrument. Virological indicators were determined using an electrochemiluminescence assay performed on cobas e 601 analyzer (Roche Laboratories, Mannheim, Germany). The detection ranges of HBsAg and anti-HBs levels were 0.05–52,000 IU/mL and 2.00–1,000 IU/L, respectively. Anti-HBs levels greater than 10 IU/L were considered positive, and levels lower than 10 IU/L were considered negative. Notably, the patients with serum levels ranging from 2 to 10 IU/L of anti-HBs indicates an need for enhanced anti-HBs response to infection (13). Semi-quantitative and quantitative assays were performed for HBeAg and hepatitis B e antibody (anti-HBe), using cut-off index (COI) and PEIU/mL as the units of quantification, respectively. The Quantitative Fluorescence Diagnostic HBV Kit (Sansure Biotech, Changsha, China) was applied to measure levels of serum HBV DNA, with a detection range of 20–5.0E+09 IU/mL. HBV genotype analysis was performed using the HBV Genotype Kit (Sansure Biotech, Changsha, China). Transient elastography, performed with an ultrasonic cirrhosis detector (ET MEDICAL Technology Co., Ltd., Shenzhen, China), was used for liver stiff measurements (LSM).

According to natural history of chronic HBV infection (14, 15), chronically HBV-infected children can be divided into four typical phases groups: patients in “immune tolerance phase” exhibited HBeAg positivity, with HBV DNA >2 × 10^7^ IU/mL and normal serum levels of ALT; patients in “immune clearance phase” exhibited HBeAg positivity, with HBV DNA >2 × 10^4^ IU/mL and elevated serum levels of ALT; patients classified as “inactive carriers” presented HBeAg negativity, HBV DNA <2 × 10^3^ IU/mL and normal serum levels of ALT; patients in “reactivation phase” exhibited HBeAg negativity, with HBV DNA >2 × 10^3^ IU/mL and elevated serum levels of ALT. Patients did not meet the above criteria were classified as being in the indeterminate grey phase.

### Statistical analysis

The IBM SPSS Statistics 25 was used to perform statistical analyses. Continuous variables were expressed using medians (SD or IQRs) while categorical variables were described using numbers and percentages. Independent *t* tests and Mann–Whitney *U* tests were, respectively, applied to test continuous variables with normal and skewed distribution, and the categorical variables were analyzed by Chi-square or Fisher’s exact test. Univariate and multivariate regression analyses were conducted to assess the associated factors with the coexistence for HBsAg and anti-HBs, with the odds ratios (*ORs*) and corresponding 95% confidence intervals (*CIs*) calculated. Two-tailed *p* value of less than 0.05 was considered as statistically significant differences.

## Results

### Clinical and biochemical characteristics of participants

**Figure 1:**
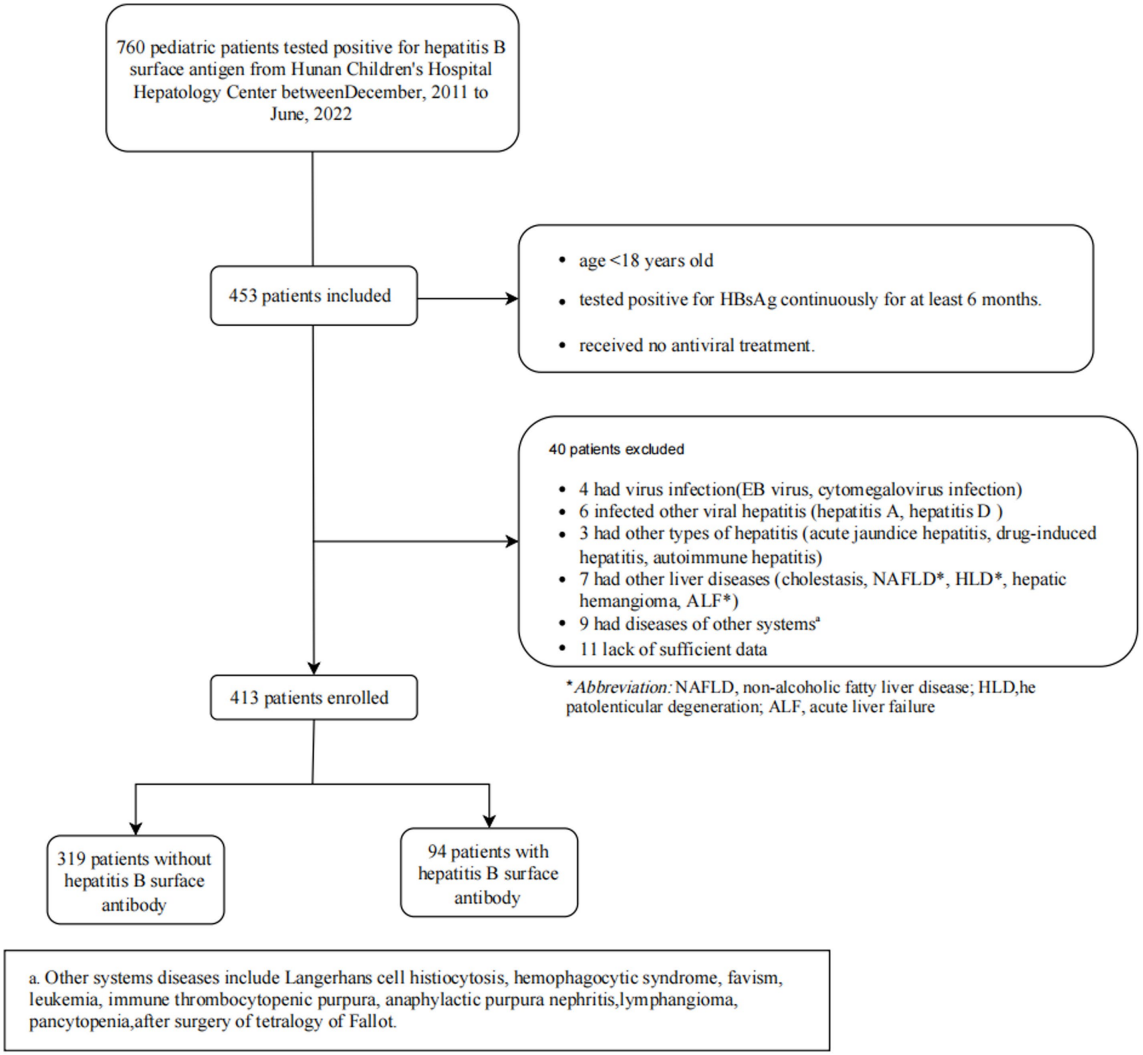
The flowchart of patient selection.

The study initially identified 760 children with Chronic HBV infection at Hunan Children’s Hospital from December 2011 to June 2022, as shown in Figure 1. 453 patients were included based on inclusion criteria. According to exclusion criteria, finally, 413 participants were enrolled. The median (IQR) age of all study participants was 4.0(3.0–8.0) years, with 270 (65.3%) male patients. A total of 94 (22.8%) patients were tested to be coexisting of HBsAg and anti-HBs. Among the patients who underwent the HBV genotype test (*n* = 295), 250 (84.7%) carried HBV genotype B and 45 (15.3%) carried genotype C.

**Figure 2:**
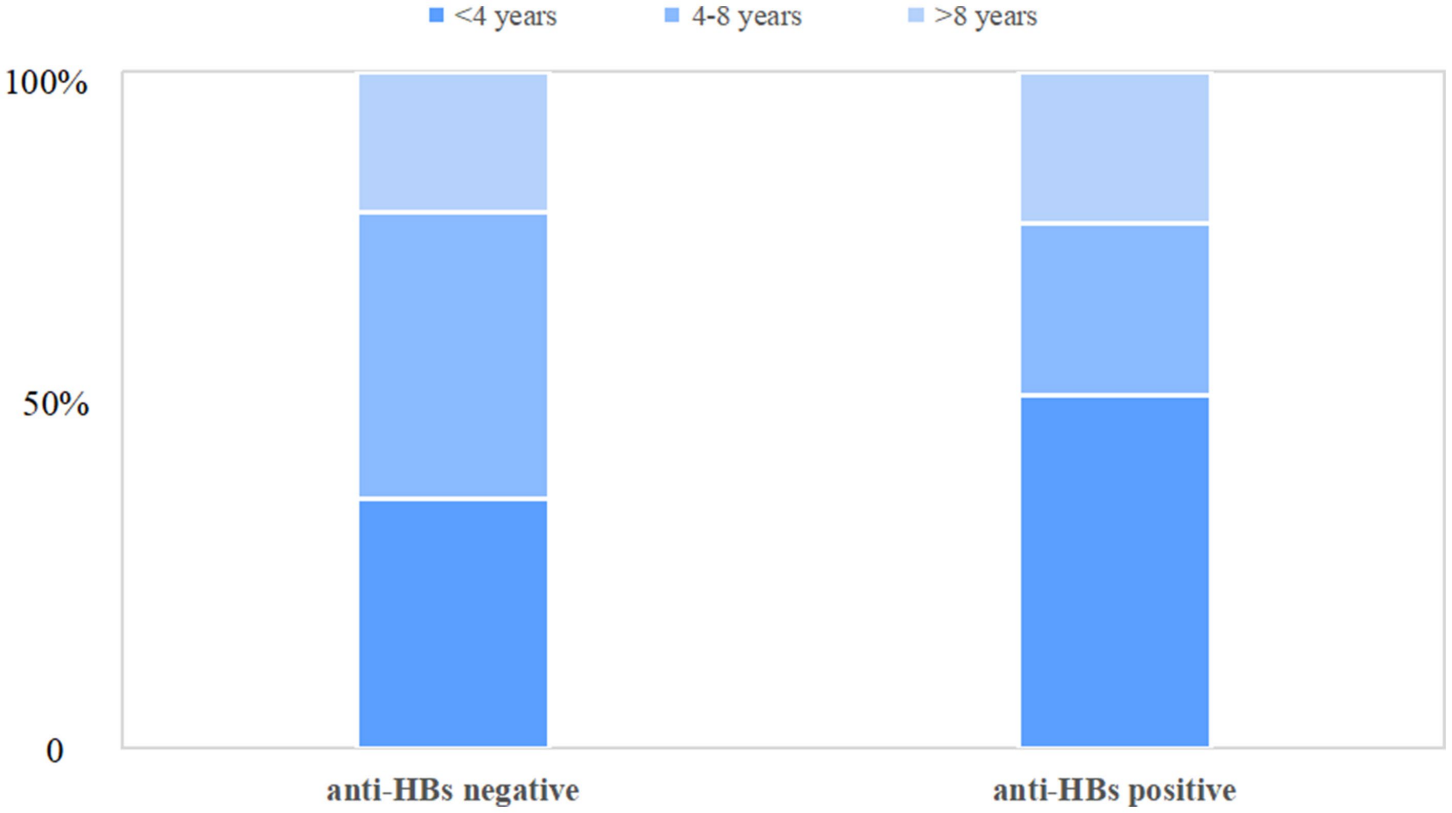
Comparison of age distribution between children with anti-HBs negative and positive.

Table 1 illustrates the clinical and biochemical indices of chronic HBV infection children with and without anti-HBs. Children with anti-HBs were found to be significantly younger (median[IQR], 3.0(2.0–7.3) years vs. 5.0(3.0–8.0) years, *p* = 0.047), with over half (52.1%) being younger than 4 years as depicted in Figure 2. Children with dual-positive for HBsAg and anti-HBs exhibited a higher ratio of A/G [median (IQR), 1.7(1.5–1.9) vs. 1.6(1.4–1.8), *p <* 0.001], higher serum levels of ALT [median (IQR), 66.7(30.8–126.6) U/L vs. 46.9(25.6–86.6) U/L, *p* = 0.008], and lower serum globulin levels [median(IQR), 24.3(22.5–27.1) g/L vs. 26.2(23.3–28.8) g/L, *p* = 0.001], and lower AST/ALT ratio [median(IQR), 0.9(0.7–1.4) vs. 1.1(0.8–1.6), *p* = 0.006], compared to only HBsAg-positive children. Nonetheless, there were no significant differences observed between the two groups regarding gender, maternal HBV infection status, vaccination status, platelet count, total protein, albumin, AST, total bilirubin, γ-glutamyl transpeptidase, alkaline phosphatase, or genotype distribution (B vs. C). Additionally, the APRI and FIB-4 scores demonstrated no significant difference between the two groups (all *p*>0.05). Of 114 patients with available LSM data, no significant difference in the degree of liver fibrosis was found between patients with or without anti-HBs (*p* = 0.300). Furthermore, an analysis of the clinical characteristics of patients with anti-HBs levels below 2 IU/L and those between 2 and 10 IU/L was performed, as detailed in Supplementary Table S1.

**Table 1:** Comparison of demographic and clinical characteristics between chronically HBV-infected children with and without anti-HBs.

Characteristic	Anti-HBs negative (anti-HBs < 10 IU/L)	Anti-HBs positive (anti-HBs > 10 IU/L)	*p* value
Total patients, No.	319	94	
Age, median (IQR), years	5.0 (3.0–8.0)	3.0 (2.0–7.3)	**0.047**
Group, years			
<4	118/319 (37.0%)	49/94 (52.1%)*^#^	**0.009**
4 ~ 8	135/319 (42.3%)	24/94 (25.5%)	
≥8	66/319 (20.7%)	21/94 (22.3%)	
Gender			
Female	111/319 (34.8%)	32/94 (34.0%)	0.893
Male	208/319 (65.2%)	62/94 (66.0%)	
Maternal HBV infection status, *n*^1^			
Yes	204/228 (89.5%)	60/65 (92.3%)	0.500
No	24/228 (10.5%)	5/65 (7.7%)	
Vaccination status, *n*^2^			
Yes	215/218 (98.6%)	60/60 (100%)	0.361
No	3/218 (1.4%)	0/60 (0%)	
PLT, median(IQR),**×**10^9/L^ULN, 3^	273.0 (224.0–321.0)	261.5 (218.5–318.8)	0.571
TP, median(IQR), g/L^ULN^	67.5 (64.9–71.0)	66.8 (63.1–70.2)	0.103
GLO, median(IQR), g/L^ULN^	26.2 (23.3–28.8)	24.3 (22.5–27.1)	**0.001**
ALB, median(IQR), g/L^ULN^	41.5 (39.2–44.1)	42.1 (39.8–44.1)	0.143
A/G, median(IQR)	1.6 (1.4–1.8)	1.7 (1.5–1.9)	**<0.001**
ALT, median(IQR), IU/L^ULN^	46.9 (25.6–86.6)	66.7 (30.8–126.6)	**0.008**
AST, median(IQR), IU/L^ULN^	50.8 (35.4–84.2)	59.3 (39.0–97.4)	0.075
AST/ALT, median(IQR)^ULN^	1.1 (0.8–1.6)	0.9 (0.7–1.4)	**0.006**
TB, median(IQR), μmol/L^ULN^	9.3 (7.1–12.5)	9.3 (7.4–12.4)	0.809
GGT, median(IQR), IU/L^ULN, 4^	12.0 (9.1–20.0)	12.0 (9.0–21.8)	0.986
AKP,median(IQR), IU/L^ULN, 5^	234.0 (189.8–279.0)	226.0 (196.8–278.5)	0.808
APRI Score, Median (IQR)^a,3^	0.5 (0.3–0.9)	0.6 (0.3–1.0)	0.082
FIB-4 Score, Median (IQR)^a,3^	0.1 (0.1–0.2)	0.1 (0.1–0.2)	0.103
Genotype^6^			
B	188/225 (83.6%)	62/70 (88.6%)	0.308
C	37/225 (16.4%)	8/70 (11.4%)	
LSM，kPa^**7**^	5.6 (4.5–6.5)	5.6 (5.0–7.3)	0.300

PLT, platelets; TP, total protein; GLO, globulin; ALB, albumin; A/G, ratio of albumin to globulin; ALT, alanine transaminase; AST, aspartate transaminase; ALT/AST, ratio of ALT to AST; TB, total bilirubin; GGT, γ-glutamyl transpeptadase; AKP, alkaline phosphatase; LSM, Liver stiff measurement.

*Compared to children aged 4–8, *p* < 0.017; ^#^compared to children older than 8, *p* > 0.017.

^a^APRI: the AST to PLT ratio index, it is calculated as the serum AST level measured in U/L divided by the upper limit of the normal AST level, then divided by the serum PLT count measured in 10^9/L, finally multiplied by 100. FIB-4: the fibrosis index based on four factors, it is calculated as the age measured in years multiplied by the serum AST level measured in U/L, then divided by the PLT count measured in 10^9/L, finally multiplied by the square root of serum ALT level measured in U/L. ^ULN^The upper limits of norm (ULN) for these clinical markers was all referenced to the specific ULN in the laboratory. Refer to Supplementary Table S4 for further information.

^**1**^This category exclude 120 patients with missing data, including 91 patients with negative for anti-HBs and 29 patients with positive for anti-HBs.

^2^This category exclude 135 patients with missing data, including 101 patients with negative for anti-HBs and 34 patients with positive for anti-HBs.

^3^This category excludes 16 patients with missing data, including 12 patients with negative for anti-HBs and 4 patients with positive for anti-HBs.

^4^This category excludes 62 patients whose blood samples were not tested for this indicator, including 48 patients with negative for anti-HBs and 14 patients with positive for anti-HBs.

^5^This category excludes 171 patients whose blood samples were not tested for this indicator, including 125 patients with negative for anti-HBs and 44 patients with positive for anti-HBs.

^6^This category excludes 118 patients whose blood samples were not tested for this indicator, including 94 patients with negative for anti-HBs and 24 patients with positive for anti-HBs.

^7^This category includes 114 patients with available liver stiff measurement data, consisting of 86 patients with negative for anti-HBs and 28 patients with positive for anti-HBs.

Bolding make it easier for reviewers to quickly spot statistically significant values.

### Virological characteristics of participants

Virological characteristics of enrolled subjects with or without anti-HBs were presented in Table 2. Children with anti-HBs demonstrated lower levels of HBsAg [median (IQR), 3.4(2.5–4.1) log_10_IU/mL vs. 4.2(3.0–4.7) log_10_IU/mL, *p <* 0.001] and HBV DNA [median (IQR), 6.5(5.3–7.3) log_10_IU/mL vs. 7.2(6.0–7.9) log_10_IU/mL, *p* < 0.001] compared to patients without anti-HBs. The patients who were double-positive for HBsAg and anti-HBs had a greater likelihood of testing positive for both HBeAg and anti-HBe [12/94 (12.8%) vs. 17/319(5.3%), *p* = 0.013]. However, no statistically significant differences were found in the status and serum levels of HBeAg and anti-HBe between the two groups (all *p* > 0.05). In addition, the virological characteristics of children with serum levels below 2 IU/L and 2–10 IU/L were also compared. Significantly, when compared to children with anti-HBs <2 IU/L, children with anti-HBs levels of 2–10 IU/L exhibited higher rates of HBeAg negativity [11/84(13.1%) vs. 14/235(6.0%), *p* = 0.037] and anti-HBe positivity [17/84(20.2%) vs. 20/235(10.6%), *p* = 0.026], with lower levels of HBeAg [median(IQR), 1390.0(369.1–1538.0) COI vs. 1475.0(1141.5–1905.0) COI, *p* = 0.002] and higher levels of anti-HBe [median(IQR), 5.6(1.8–7.0) COI vs. 6.2(4.8–8.5), *p* = 0.003], as shown in Supplementary Table S2.

**Table 2:** Comparison of virological characteristics between chronically HBV-infected children with and without anti-HBs.

Characteristic	Anti-HBs negative (*n* = 319)	Anti-HBs positive (*n* = 94)	*p* value
HBsAg, median(IQR), log_10_IU/mL	4.2 (3.0–4.7)	3.4 (2.5–4.1)	**<0.001**
Anti-HBs, median(IQR), IU/L	2.0 (2.0–2.1)	34.7 (17.5–110.3)	**<0.001**
HBeAg status			
Positive	294/319 (92.2%)	87/94 (92.6%)	0.901
Negative	25/319 (7.8%)	7/94 (7.4%)	
HBeAg, median(IQR), COI^1, #^	1453.0 (1039.5–1814.0)	1427.5 (528.9–1853.5)	0.370
anti-HBe status			
Positive	42/319 (13.2%)	19/94 (20.2%)	0.091
Negative	277/319 (86.8%)	75/94 (79.8%)	
anti-HBe, median(IQR), COI^1, *^	6.0 (3.9–7.9)	5.8 (2.3–7.9)	0.325
Coexistent HBeAg and anti-HBe	17/319 (5.3%)	12/94 (12.8%)	**0.013**
HBV-DNA, median, log_10_IU/mL^2^	7.2 (6.0–7.9)	6.5 (5.3–7.3)	**<0.001**

HBsAg, hepatitis B surface antigen; anti-HBs, hepatitis B surface antibody; HBeAg, hepatitis B e antigen; anti-HBe, hepatitis B e antibody.

^#^Serum levels of HBeAg: negative, 0–1 COI; positive, >1 COI.

^*^Serum levels of anti-HBs: negative > 1 COI; positive < 1 COI.

^1^This category excludes 56 patients whose blood samples were tested by quantitative assays, in the units of PEIU/mL, including 50 patients with negative for anti-HBs and 6 patients with positive for anti-HBs.

^2^This category excludes 17 patients with missing data, including 10 patients with negative for anti-HBs and 7 patients with positive for anti-HBs.

Bolding make it easier for reviewers to quickly spot statistically significant values.

### Distribution of chronic HBV infection

Among 413 enrolled patients, 78 (18.8%) were in the immune tolerance phase, 195 (47.2%) were in the immune clearance phase, 12 (2.9%) inactive carriers and 16 (3.9%) were in the reactivation phase, and 112 (27.1%) did not meet the diagnostic criteria according to natural history of chronic HBV infection, regarding as indeterminate grey phase. According to Supplementary Table S3, the majority of children with or without anti-HBs were in the immune clearance phase, while children with anti-HBs were observed to have a higher likelihood of being in the immune tolerance phase compared to those without anti-HBs. There were significantly distributional differences in the natural history between the two groups (*p* < 0.05).

### Associated features of chronically HBV-infected children with coexistence of HBsAg and anti-HBs

In the univariate analysis (illustrated in Table 3), several factors showed significant association with the co-positivity of HBsAg and anti-HBs, including globulin levels (*OR*, 0.91, 95%*CI*, 0.85–0.96; *p* = 0.002), AST/ALT ratios (*OR*, 0.64, 95%*CI*, 0.42–0.96; *p* = 0.031), serum HBsAg (*OR*, 0.60, 95%*CI*, 0.48–0.74; *P*<0.001), HBV DNA (*OR*, 0.82, 95%*CI*, 0.72–0.94; *p* = 0.004) and coexistent HBeAg and anti-HBe (*OR*, 2.60, 95%*CI*, 1.19–5.66; *p* = 0.016). The results of multivariate analysis showed that the levels of serum globulin [*OR*, 0.88, 95%*CI*, 0.83–0.94; *p<*0.001], AST/ALT ratios [*OR*, 0.55, 95%*CI*, 0.35–0.88; *p* = 0.012] and HBsAg levels [*OR*, 0.59, 95%*CI*, 0.46–0.76; *p<*0.001] remained significantly associated with this special serological state.

**Table 3:** Univariate and multivariate analysis of the coexistence of HBsAg and anti-HBs.

	Univariate analysis		Multivariate analysis	
Variable	OR(95%CI)	*p* value	OR(95%CI)	*p* value
Sex				
Female	1 (Reference)	0.893	NA	NA
Male	1.03 (0.64–1.68)		NA	NA
Age	0.95 (0.89–1.02)	0.139	NA	NA
TP	0.96 (0.92–1.01)	0.119	NA	NA
ALB Level	1.06 (0.99–1.13)	0.125	NA	NA
GLO Level	0.91 (0.85–0.96)	**0.002**	0.88 (0.83–0.94)	**<0.001**
ALT level	1.00 (1.00–1.00)	0.349	NA	NA
AST level	1.00 (1.00–1.00)	0.578	NA	NA
AST/ALT	0.64 (0.42–0.96)	**0.031**	0.55 (0.35–0.88)	**0.013**
HBsAg level(Log_10_)	0.60 (0.48–0.74)	**<0.001**	0.59 (0.45–0.76)	**<0.001**
HBV-DNA level(log_10_)	0.82 (0.72–0.94)	**0.004**	0.96 (0.81–1.15)	0.668
HBeAg Status				
Negative	1 (Reference)	0.901	NA	NA
Positive	1.06 (0.44–2.53)		NA	NA
anti-HBe Status				
Negative	1 (Reference)	0.093	NA	NA
Positive	1.67 (0.92–3.04)		NA	
Coexistent HBeAg and anti-HBe				
No	1(Reference)	**0.016**	1 (Reference)	0.078
Yes	2.60 (1.19–5.66)		2.31 (0.91–5.88)	

Bolding make it easier for reviewers to quickly spot statistically significant values.

## Discussion

This retrospective study assessed the prevalence of coexistence of HBsAg and anti-HBs in CHB children with chronic HBV infection and investigated the clinical and virological features of patients with this specific serological pattern in children population. In our study, a considerable prevalence of coexistence of HBsAg and anti-HBs in children with chronic HBV infection was reported, reaching up to 22.8% (94/414). This prevalence was significantly higher than the rates reported in previous studies involving adult patients (4, 5, 7, 10, 12, 16, 17). Moreover, chronically HBV-infected children with coexistence of HBsAg and anti-HBs had a younger age and higher serum ALT levels and rates of coexistent HBeAg and anti-HBe, and lower serum levels of globulin, HBsAg, HBV DNA, and AST/ALT ratio.

The prevalence of this specific serological pattern in patients with chronic HBV infection, based on numerous studies conducted in the past decade (4, 5, 7, 10, 12, 16, 17), was generally less than 10%. According to a large-scale hospital-based study conducted in China, among 6,534 patients with HBV infection and a median age of 41, 4.2% (277 of 6,534) patients were simultaneously positive for HBsAg and anti-HBs (7). Similarly, an American study including 1,462 participants (age range 4–80) also reported a low prevalence of 1.2% for coexistence of HBsAg and anti-HBs (10). Apparently, the prevalence of 22.8% observed in the current investigation was significantly higher than the rates mentioned in previous studies which mostly enrolled adult CHB patients characterized by relatively long disease courses. The discovery of this specific serological pattern could be traced back as early as 1976 (18), while the reason for such difference in concurrently positive for HBsAg and anti-HBs is enigmatic. Firstly, prior studies (19–22) have proved the relationship between persistent detectability of anti-HBs and escape mutants of HBsAg caused by variations in key regions of HBV genome. Therefore, genetic difference of HBV in various races and regions may contribute to explain the variance in prevalence. Secondly, the immunological characteristics of children could be another explanation for the elevated prevalence of this serological pattern in chronically HBV-infected children. Throughout the natural history of chronic HBV infection, anti-HBs plays a pivotal role in the dynamic seroconversion of HBsAg and reflects the immune capacity against HBV infection (18, 23). Continuous detectability of anti-HBs serves as an indicator of HBsAg clearance. However, the anti-HBs failed to fully neutralize HBsAg in certain individuals (12, 16, 24), especially in those with weak immunity such as those with immunosuppression, or underdeveloped immune system. In current study, the young patients had a median age of 4.0 (3.0–8.0) years and those with anti-HBs were younger, potentially indicating an immature immunity. In the immune clearance phase, HBsAg might not be eliminated completely by adequate homologous antibodies, even though the patients were vaccinated. Furthermore, host immune responses, especially specific immune cell, may contribute to a high expression of anti-HBs titres in treatment-naive patients, for the crucial function in the virus clearance process (2, 25). Additionally, as a crucial preventative measure against mother-to-child transmission from mothers with high viral load and positive HBeAg, hepatitis B vaccination can induce the production of protective anti-HBs while enhancing gene mutation in the HBsAg region. Therefore, high vaccination rates may account for such high prevalence of double positive for HBsAg and anti-HBs (23). Moreover, the effect of the vaccine diminishes or even disappears over time, leading to a lower probability of the serological pattern persisting with age. The decreased prevalence of coexistent HBsAg and anti-HBs in the adult population supports this hypothesis. But more comprehensive validation is needed in the future study.

The coexistence of positive HBsAg and corresponding antibody was considered an indicator of advanced hepatic disease, including liver fibrosis and cirrhosis in patients with CHB (9, 10, 24, 25). In current study, the comparison of clinical factors between chronically HBV-infected children with and without anti-HBs indicated significantly higher levels of serum ALT in patients who were anti-HBs positive. Furthermore, the ratio of AST/ALT were found to be independently correlated with this coexistent serological pattern, suggesting more intensive hepatocyte injury among children with anti-HBs (26). In addition, the levels of globulin in both groups were within the normal range, and the levels of ALT in both groups were subtly higher than the upper limits of normal (ULN) according to clinical diagnostic criteria (26). Generally, globulin is considered an indicator of liver function and a diagnostic indicator of cirrhosis associated with CHB. However, in the early process of chronic HBV infection, relying solely on the biochemical indices that are usually within normal range may not effectively recognize the status of immunogenesis and liver pathogenesis. Therefore, according to the correlation between coexistent positivity for HBsAg and anti-HBs and early liver impairment, the emergence of this specific serological pattern could serve as a remarkable signal for monitoring the development of liver impairment in children.

In the comparison of virological indicators between chronically HBV-infected children with and without anti-HBs, we found that patients with anti-HBs exhibited lower serum levels of HBsAg and HBV DNA compared to those without anti-HBs. The quantitative levels of HBsAg were positively correlated with HBV DNA, which is known as an index for evaluating HBV replication activity (27, 28). Additionally, higher rates of coexistent HBeAg and anti-HBe also demonstrated that more patients were in the stage of natural seroclearance of HBeAg. Therefore, this serological pattern might indicate relatively low levels of HBV replication, suggesting the protective function of anti-HBs to neutralize HBsAg and clear corresponding viral particles. However, the majority of the children had HBV DNA levels exceeding 1 million IU/mL, meeting the criteria for continuous surveillance and antiviral treatment according to the *guidelines for the prevention and treatment of chronic hepatitis B* (version 2022). These findings were consistent with the results reported by Liu (12), but differed from the results revealed by Wang et al. (7) and Colson et al. (24), of whose researches demonstrated that coexistence of HBsAg and anti-HBs was related to higher load of HBV DNA. The study of Liu Y showed a constant detectability of serum HBV DNA and HBsAg in children with coexisting HBsAg and corresponding antibody during the period of follow-up, suggesting the disability of anti-HBs to eliminate HBV infection completely. Other studies (7, 24) also revealed the similar results. Therefore, continuous monitoring of serological and virological indices in children with chronic HBV infection is needed to certify whether HBsAg-positive chronic infection accompanied by positivity for anti-HBs is predisposed to insufficient neutralizing capacity of anti-HBs in future study.

### Limitations

This study has several limitations. First, we did not perform the detection of the gene coding sequence or the determination of the HBsAg sub-determinants to investigate the frequency of immune escape or verify their association with coexistent HBsAg and anti-HBs, apart from the genotype of HBV. Another major limitation is the complex and long-term interaction among the host, virus, and environment during the natural history of chronic HBV infection, leading to constant changes in serological indices (29–31). Therefore, the results obtained from a single time point cannot reflect the complete characteristics of the entire coexisting period. To obtain consistent evidence in continuously changing biochemical and virological indices, the study should be validated with additional follow-up data. Lastly, a limitation of this study is the regional bias, because most of the enrolled patients are from Hunan Province. As shown in the previous research (32) of Xia, et al., the seroprevalence of HBsAg in Hunan was 0.82% among 1-4-year children and 1.71% among 5-14-year children. The prevalence of HBsAg in children aged 1–4 years is mildly higher than the national prevalence of the same age group (0.6% in children under 5) during a similar time period (33). Therefore, regional difference of seroprevalence of HBsAg are indispensable biases to studies that inquiry the prevalence and clinical features of general population based on collected samples.

## Conclusion

In summary, this retrospective study showed a considerable prevalence of coexistence of HBsAg and anti-HBs in chronic HBV-infected children. Children who exhibit a specific serological pattern characterized by higher ALT levels, lower HBsAg levels, and lower HBV DNA levels may be at a higher risk of hepatic injury and have reduced HBV replication activity. Additionally, these children tend to be younger, further emphasizing the importance of understanding this unique serological profile in pediatric patients. Close monitoring of the related biochemical and virological indices is necessary in chronically HBV-infected children with anti-HBs to assess the progression of liver injury and HBV replication in the early stage of chronic HBV infection.

## Data availability statement

The raw data supporting the conclusions of this article will be made available by the authors, without undue reservation.

## Ethics statement

The studies involving humans were approved by XiangYa School of Public Health Central South University. The studies were conducted in accordance with the local legislation and institutional requirements. The human samples used in this study were acquired from the routine tests when the patients visited. Written informed consent for participation was not required from the participants or the participants’ legal guardians/next of kin in accordance with the national legislation and institutional requirements.

## Author contributions

YX: Formal analysis, Methodology, Visualization, Writing – original draft. ShL: Data curation, Investigation, Resources, Supervision, Writing – review & editing. WO: Data curation, Resources, Supervision, Writing – review & editing. ZY: Formal analysis, Investigation, Writing – review & editing. XL: Investigation, Supervision, Writing – review & editing. YG: Investigation, Supervision, Writing – review & editing. MY: Data curation, Investigation, Writing – review & editing. LY: Investigation, Writing – review & editing. SiL: Investigation, Writing – review & editing. SP: Conceptualization, Methodology, Writing – review & editing, Data curation, Funding acquisition, Supervision.
