# Supplementary material for: Clinical and virological characteristics of coexistent hepatitis B surface antigen and antibody in treatment-naive children with chronic hepatitis B virus infection
Source: Front Public Health. 2024 Jun 17;12:1380771. doi: 10.3389/fpubh.2024.1380771 (PMC11215143; doi:10.3389/fpubh.2024.1380771)
Supplement: Supplementary file 1 [file Table_1.DOCX]

| **TableS1 Comparison of demographic and clinical characteristics of chronic HBV-infected children with anti-HBs below 2 IU/L and between 2-10 IU/L** | | | |
| --- | --- | --- | --- |
| **Characteristic** | **anti-HBs <2 IU/L** | **anti-HBs 2-10 IU/L** | ***P* value** |
| **Total patients, No.** | 235 | 84 |  |
| **Age, median (IQR), years** | 5.0(3.0-8.0) | 4.5(3.0-8) | 0.684 |
| **Gender** |  |  |  |
| Female | 78/235(33.2%) | 33/84(39.3%) | 0.314 |
| Male | 157/235(66.8%) | 51/84(60.7%) |  |
| **Maternal HBV infection status, n^1^** | | | |
| Yes | 148/169(87.6%) | 56/59(94.9%) | 0.114 |
| No | 21/169(12.4%) | 3/59(5.1%) |  |
| **Vaccination status, n^2^** | | | |
| Yes | 162/164(98.8%) | 53/54(98.1%) | 1.000 |
| No | 2/164(1.2%) | 1/54(1.9%) |  |
| **PLT, median(IQR),**×**10^9/L^3^** | 274.5(224.0-322.0) | 272.0(224.0-320.0) | 0.776 |
| **TP, median(IQR),g/L** | 67.9(64.9-71.0) | 66.8(64.8.1-70.8) | 0.409 |
| **GLO, median(IQR),g/L** | 26.2(23.3-29.0) | 26.0(23.2-28.0) | 0.603 |
| **ALB, median(IQR),g/L** | 41.4(39.1-44.2) | 41.6(39.3-43.4) | 0.856 |
| **A/G, median(IQR)** | 1.6(1.4-1.8) | 1.6(1.5-1.8) | 0.519 |
| **ALT, median(IQR),IU/L** | 46.0(24.6-83.0) | 52.3(27.8-122.6) | 0.088 |
| **AST, median(IQR),IU/L** | 48.9(33.8-80.2) | 55.1(39.7-104.1) | **0.015** |
| **AST/ALT, median(IQR)** | 1.1(0.8-1.6) | 1.1(0.8-1.5) | 0.832 |
| **TB, median(IQR), μmol/L** | 9.2(7.1-12.9) | 9.8(7.3-12.2) | 0.842 |
| **GGT, median(IQR), IU/L^4^** | 12.6(9.5-21.5) | 11.6(9.0-17.4) | 0.331 |
| **AKP ,median(IQR), IU/L5** | 229.0(182.2-262.8) | 260.0(198.8-304.8) | **0.032** |
| **APRI Score, Median (IQR)^a,3^** | 0.4（0.3-0.8） | 0.6（0.3-1.2） | 0.055 |
| **FIB-4 Score, Median (IQR)^a,3^** | 0.1（0.1-0.2） | 0.1（0.1-0.2） | 0.572 |
| **Genotype^6^** |  |  |  |
| B | 130/154(84.4%) | 58/71(81.7%) | 0.608 |
| C | 24/154(1.56%) | 13/71(18.3%) |  |
| **LSM，kPa^7^** | 5.5(4.4-6.5) | 5.9(4.9-6.6) | 0.384 |

^1^ This category exclude 91 patients with missing data, including 66 patients with anti-HBs<2 IU/L and 25 patients with anti-HBs between 2-10 IU/L.

^2^ This category exclude 101 patients with missing data, including 71 patients anti-HBs<2 IU/L and 30 patients with anti-HBs between 2-10 IU/L.

^3^ This category excludes 12 patients with missing data, including 11 patients with anti-HBs<2 IU/L and 1 patients with anti-HBs between 2-10 IU/L.

^4^ This category excludes 48 patients whose blood samples were not tested for this indicator, including 42 patients with anti-HBs<2 IU/L and 6 patients with anti-HBs between 2-10 IU/L.

^5^ This category excludes 127 patients whose blood samples were not tested for this indicator, including 95 patients with anti-HBs<2 IU/L and 32 patients with anti-HBs between 2-10 IU/L.

^6^ This category excludes 94 patients whose blood samples were not tested for this indicator, including 81 patients with anti-HBs<2 IU/L and 13 patients withanti-HBs between 2-10 IU/L.

^7^ This category includes 86 patients with available liver stiff measurement data, consisting of 52 patients with anti-HBs<2 IU/L and 34 patients with anti-HBs levels 2-10 IU/L.

| **Table S2 Comparison of virological characteristics of chronic HBV-infected children with anti-HBs below 2IU/L and between 2-10IU/L** | | | |
| --- | --- | --- | --- |
| **Characteristic** | **Anti-HBs <2 IU/L**  **(n=235)** | **anti-HBs 2-10 IU/L**  **(n=84)** | ***P* value** |
| **HBsAg, median(IQR), log_10_IU/mL** | 4.3(2.7-4.7) | 4.1(3.2-4.6) | 0.167 |
| **HBeAg Status** |  |  |  |
| positive | 221/235(94.0%) | 73/84(86.9%) | **0.037** |
| negative | 14/235(6.0%) | 11/84(13.1%) |  |
| **HBeAg, median(IQR), COI^#^** | 1475.0  (1141.5-1905.0) | 1390.0  (369.1-1538.0) | **0.002** |
| **anti-HBe Status** |  |  |  |
| positive | 20/235(10.6%) | 17/84(20.2%) | **0.026** |
| negative | 210/235(89.4%) | 67/84(79.8%) |  |
| **anti-HBe, median(IQR),COI^#^** | 6.2(4.8-8.5) | 5.6(1.8-7.0) | **0.003** |
| **Coexistence of HBeAg and anti-HBe** | 11/235(4.7%) | 6/84(7.1%) | 0.562 |
| **HBV-DNA, median, l og_10_IU/mL*** | 7.2(6.1-7.9) | 7.3(5.1-7.9) | 0.308 |

^#^ This category excludes 50 patients whose blood samples were tested by quantitative assays, in the units of PEIU/mL, including 49 patients with anti-HBs<2 IU/L and 1 patients with anti-HBs between 2-10 IU/L.

* This category excludes 10 patients with missing data, including 9 patients with anti-HBs<2 IU/L and 1 patients with anti-HBs between 2-10 IU/L.

| **Table S3 Comparison of phases of chronic HBV infection between chronically HBV-infected children with and without anti-HBs** | | | |
| --- | --- | --- | --- |
| **Phases of chronic HBV infection** | **Anti-HBs negative**  **(n=319)** | **Anti-HBs positive**  **(n=94)** | ***P* value** |
| Immune tolerance phase | 72/319(22.6%) | 6/94(6.4%) | **0.002** |
| Immune clearance phase | 145/319(45.5%) | 50/94(53.2%) |  |
| Inactive carriers phase | 10/319(3.1%) | 2/94(2.1%) |  |
| Reactivation phase | 13/319(4.1%) | 3/94(3.2%) |  |
| Indeterminate grey phase | 79/319(24.8%) | 33/94(35.1%) |  |

##### Table S4

##### Normal range of biochemical markers based on laboratory exam

Affiliation: Hunan Children’s Hospital

| **Indices** | **Units** | **ULN*** | **Reference** |
| --- | --- | --- | --- |
| **Platelets** | ×10^9/L | 400 | 100-400 |
| **Total protein** | g/L | 80 | 55-80 |
| **Albumin** | g/L | 55.00 | 35.00-55.00 |
| **Globulin** | g/L | 35 | 20-35 |
| **Alanine transaminase** | IU/L | 40 | 0-40 |
| **Aspartate transaminase** | IU/L | 40 | 0-40 |
| **Total bilirubin** | μmol/L | 17.0 | 3.4-17.0 |
| **Direct bilirubin** | μmol/L | 6.0 | 0-6.0 |
| **Indirect bilirubin** | μmol/L | 17 | 3.0-17.0 |
| **γ-glutamyl transpeptadase** | IU/L | 50 | 0-50 |
| **Alkaline phosphatase** | IU/L | 500 | <500^#^ |

* ULN：upper limits of norm

# The reference range for alkaline phosphatase in children aged 12 years or below is <500 IU/L, in male children aged 12-15 years is <750 IU/L.
